# Type 2 diabetes in South Asians: similarities and differences with white Caucasian and other populations

**DOI:** 10.1111/j.1749-6632.2012.06838.x

**Published:** 2013-01-14

**Authors:** Unjali P Gujral, R Pradeepa, Mary Beth Weber, KM Venkat Narayan, V Mohan

**Affiliations:** 1 Graduate Division of Biological and Biomedical Sciences, Nutrition and Health Sciences Program, Laney Graduate School, Emory UniversityAtlanta, Georgia; 2 Madras Diabetes Research Foundation and Dr. Mohan's Diabetes Specialities Centre, WHO Collaborating Centre for Noncommunicable Diseases, Prevention and ControlChennai, India; 3 Hubert Department of Global Health and Epidemiology, Rollins School of Public Health, Emory UniversityAtlanta, Georgia, USA

**Keywords:** type 2 diabetes mellitus, South Asians, prevalence, ethnic comparison

## Abstract

Type 2 diabetes mellitus (T2DM) is one of the leading causes of morbidity and mortality. While all ethnic groups are affected, the prevalence of T2DM in South Asians, both in their home countries and abroad, is extremely high and is continuing to rise rapidly. Innate biological susceptibilities coupled with rapid changes in physical activity, diet, and other lifestyle behaviors are contributing factors propelling the increased burden of disease in this population. The large scope of this problem calls for investigations into the cause of increased susceptibility and preventative efforts at both the individual and population level that are aggressive, culturally sensitive, and start early. In this review, we outline the biological and environmental factors that place South Asians at elevated risk for T2DM, compared with Caucasian and other ethnic groups.

## Introduction

Type 2 diabetes mellitus (T2DM) currently affects approximately 366 million people worldwide.^1^ This includes individuals in developed countries, but also those living in urban and rural areas of developing countries.^2^–^4^ South Asians (those who live in or have their roots in India, Pakistan, Sri Lanka, Bangladesh, Nepal, Bhutan, or the Maldives)^2^ seem to be at especially high risk for developing T2DM. While the overall prevalence of T2DM in South Asia is high and increasing, there is considerable heterogeneity across South Asian countries (Table 1). Much of this heterogeneity can be attributed to differing states of socioeconomic development, variations in lifestyle factors, and differences in prevalence of undiagnosed versus diagnosed diabetes among countries. The majority of T2DM data from South Asia have come from India, the South Asian country with the largest diabetes burden, and where the prevalence has increased steadily over the past 40 years.^2^ The most recent national prevalence study collected data from three states and one union territory covering a population of over 200 million people. The overall weighted prevalence was 10.4% in Tamilnadu, 8.4% in Maharashtra, 5.3% in Jharkhand, and 13.6% in Chandigarh.^5^ If extrapolated nationwide, these estimates translate to 62.4 million individuals in India currently living with T2DM.^5^

**Table 1:** South Asian diabetes estimates for 2011 by country

Country	Diabetes cases	National prevalence of diabetes (%)
Bangladesh	8,406,000	9.6
Bhutan	21,000	4.9
India	61,258,000	8.31
Maldives	15,000	7.6
Nepal	488,000	3.0
Sri Lanka	1,078,000	7.8

Source: International Diabetes Federation.^3^

**Figure 1:**
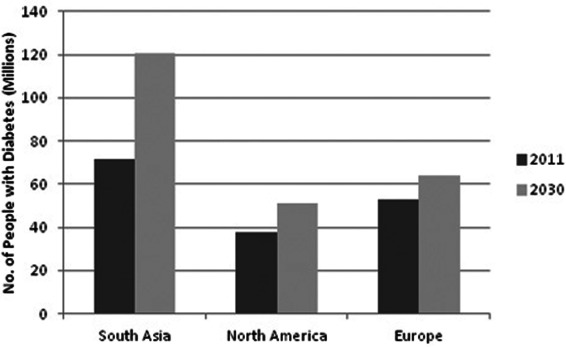
Projected number of people with diabetes (millions) by year—2011 and 2013.

**Figure 2:**
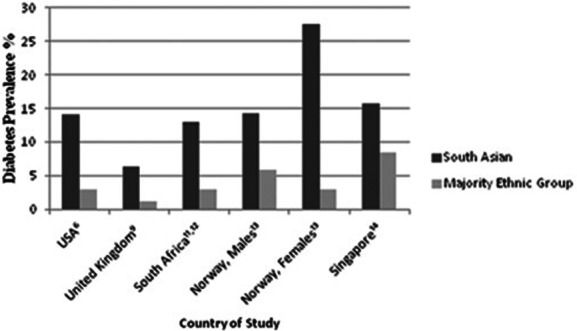
Differences in prevalence among South Asians and other ethnic groups.

By 2030 it is projected that there will be 120.9 million people with diabetes in South Asia (90–95% of these cases will be T2DM),^3^ more than double the number affected in North America or Europe (Fig. 1). The prevalence of T2DM is also high among South Asian migrant populations, with several studies noting a higher prevalence of T2DM in migrant South Asians than in other ethnic groups in the host countries.^6^–^14^ Nationally, representative studies in the United States have shown that regardless of BMI classification, South Asians have the highest BMI-specific prevalence of T2DM among all ethnic groups.^7^ A recent randomized population based study of South Asians in the United States reported an overall T2DM prevalence of 17.4% in South Asian adults. These prevalence estimates greatly exceed those in non-Hispanic whites (7.8%), non-Hispanic blacks (13%), and Hispanic Latinos (10.2%).^8^ Similar patterns have also been observed in other diaspora countries, including the United Kingdom, Fiji, South Africa, Norway, and Singapore (Fig. 2).^9^–^14^ Furthermore, although the data are limited, it appears that T2DM incidence is much higher in South Asians compared with groups in Europe and the United States (20.2 per 1,000 person-years in a study in India^15^ compared with 6.9 per 1,000 person-years in the United States,^16^ and 7.6 and 10.8 cases per 1,000 person-years for studies in Italy^17^ and Spain,^18^ respectively).

The higher prevalence and incidence of T2DM in South Asians populations worldwide is likely indicative of underlying biological factors in South Asians, coupled with recent rapid changes in dietary, activity, and other lifestyle behaviors. Herein, we discuss the current literature comparing South Asians with other populations with regard to biological (e.g., pathophysiology and genetics) and environmental factors (e.g., lifestyle) associated with T2DM. A synopsis of the paper is presented in Table 2.

**Table 2:** Comparison of diabetes risk factors among South Asians, Caucasians, and other ethnic groups

Pathophysiology	
• Insulin resistance	• Insulin resistance is a pathophysiological factor and precursor for diabetes in all ethnic populations. However, South Asians are shown to be shown to be more insulin resistant than Caucasian populations even at younger ages and lower levels of BMI. Some of this increased propensity for insulin resistance in South Asians might be attributed to greater deposition of visceral fat in South Asians as compared to Caucasians.
• Pancreatic β-cell function	• While declines in pancreatic β-cell function are involved in diabetes pathogenesis for all ethnic groups, there may be ethnic differences in the timing and relative contribution of β-cell decline. Preliminary data from South Africa indicates an early impairment of β-cell function as an underlying pathophysiological abnormality in South Asians. However, the complete mechanistic pathway determining T2DM in South Asians as compared to other ethnic groups remains unclear, and early declines in β-cell function are an important pathophysiological factor to investigate.
Risk Factors	
• Genetics	• Approximately 60 genes have been implicated in T2DM development;^43^,^44^ however, most studies were conducted in European populations. While the actual genes established may not be different, some differences exist in allele frequencies and polymorphisms.
• Age	• Increasing age elevates T2DM risk in all populations; however, South Asians develop the disease at younger ages. Mean age at diagnosis was lowest in South Asians (49 years), followed by Chinese (55 years), Blacks (57 years), and Whites (58 years) living in Canada.^57^ To date there have been no studies directly comparing the age-specific prevalence and incidence of diabetes among South Asians living in South Asia and members of other ethnic groups living in the United States and Europe.
• Anthropometry	• Higher BMI, overweight, and obesity are associated with increased risk for diabetes in all ethnic groups. However, despite similar levels of BMI, South Asians have more total abdominal and visceral fat compared to Caucasians.^33^,^58^ These differences are seen even in childhood and early adolescence.^61^–^63^
	• Prospective studies from South Asia indicate that participants who were underweight as children have a higher prevalence of overweight and obesity as adults.^67^ Furthermore, in South Asians with hyperglycemia in adulthood, a relationship was seen with low BMI before age 2, followed by accelerated increases in BMI throughout adulthood.^68^ Being that 40% of the world's low-birth-weight babies are born in India,^65^ this is an important area for future research.
• Biomarkers	• ROS, leptin, and C-reactive protein (CRP) have all been associated with an increased risk for diabetes, while adiponectin has been associated with a decreased risk.^51^,^74^–^77^ Compared to Caucasians, South Asians have exhibited elevated levels of leptin and CRP and decreased levels of adiponectin.^75^–^78^ However, to our knowledge, there have been no studies assessing levels of oxidative stress in South Asians.
• Physical inactivity	• Reductions in physical activity increase the risk of diabetes in all ethnic groups; however, South Asians appear to be even less physically active than their Caucasian counterparts.^85^,^86^ South Asians have demonstrated physical activity levels that were 50–75% lower than those of Europeans.^87^
• Diet	• Higher intakes of refined carbohydrates, saturated fats, and trans fats have been shown to increase diabetes risk in all populations, while low glycemic index foods and foods high in dietary fiber have been shown to decrease the risk.^89^,^90^ In general, a typical South Asian meal has a higher caloric intake and a higher percentage of carbohydrate than does a European meal.^92^ In South Asians who have migrated to Western countries, acculturation has led to an increased consumption of foods, such as potato chips and colas, and a decrease in consumption of fiber.^95^,^96^
• Tobacco use	• Tobacco use increases diabetes risk in all populations.^100^–^102^ While there is a higher prevalence of tobacco use in South Asian than in the United States and Europe,^104^–^107^ there are no studies directly comparing the relationship between diabetes risk and smoking in South Asians as compared to Caucasians.
• Sleep	• Studies have shown that sleep duration above or below average or disruptions in sleep can increase the risk for diabetes.^108^–^110^ However, to date, no comparative studies have been conducted on duration of sleep and or/sleep disruptions in South Asians as compared to other ethnic groups.
• Persistent organic pollutants	• Persistent organic pollutants are of recent concern regarding their association with diabetes risk.^114^ While studies in the United States have noted an association between exposure to POPs and elevated risk for diabetes,^55^ no such studies have been conducted in South Asia. However, there is some evidence of high levels of POP contaminants in South Asian waters.^116^

## Biological risk factors for T2DM

### Pathophysiology

While some studies note that the pathogenesis of T2DM begins with adiposity-induced insulin resistance followed by a subsequent decline in pancreatic β-cell function,^19^–^21^ other data point to the role of earlier declines in β-cell function, suggesting that this may be the primary defect predisposing individuals and populations to T2DM.^22^–^26^ It is possible that variances in both the propensity to develop insulin resistance as well as the compensatory ability of β cells may explain some ethnic differences in T2DM susceptibility.^27^–^30^ There is strong evidence to suggest that South Asians are more insulin resistant than Caucasians, even at younger ages and comparative levels of BMI. A study by Mohan *et al.* demonstrated increased insulin levels in South Asians as compared to Europeans, both with and without diabetes.^31^ A later study from Europe found South Asians with T2DM to be significantly more insulin resistant then their European counterparts, despite their younger age,^32^ while studies from the United States have noted a significantly lower insulin sensitivity in South Asians as compared to Caucasians, regardless of the level of total body fat.^33^

It appears that some of the increased propensity for South Asians to develop insulin resistance could be attributable to greater accumulation of visceral fat.^34^,^35^ Data from a subset of the Chennai Urban Rural Epidemiology Study (CURES) indicated that a progressive increase in visceral fat was associated with increasing glucose intolerance in South Asians.^35^ Furthermore, when compared to Caucasians, South Asians demonstrate significantly lower glucose disposal while also exhibiting significantly greater total abdominal and visceral fat.^33^ Recent studies have shown that even in nondiabetic South Asians, greater visceral fat is associated with increased insulin resistance.^36^ However, It is possible that gender differences may exist regarding body fat distribution and insulin resistance. A recent study comparing young South Asian and Caucasian women matched for BMI noted no differences in body fat distribution and insulin sensitivity between groups.^37^ While this study introduced novel findings, the sample size was fairly small, and all participants were healthy, young (mean age 24), and premenopausal. Therefore, generalizations cannot be made about women of all ages and BMI categories, and further studies exclusive to South Asian women are warranted.

In addition to an increased propensity for insulin resistance, South Asians may also experience early declines in β-cell function, compared with other ethnic groups. A study examining both insulin resistance and β-cell function in a group of East Asians, South Asians, Blacks, and Caucasians found that despite being matched on lifestyle factors and BMI the prevalence of insulin resistance in South Asian men was threefold to fourfold greater than lean men of other ethnic groups.^38^ Upon assessment of β-cell function in a subgroup of South Asian and Caucasian men it was observed that South Asians had a 30% increase in basal β-cell responsiveness; however, this increase in β-cell function was not sufficient to compensate for the degree of insulin resistance, as shown by a 60% reduction in disposition index (a measure of β-cell response to insulin resistance), in South Asian men.^38^ Additionally, a prospective study of Asian Indians in South Africa with impaired glucose tolerance (IGT) reported that, compared with controls, participants with IGT exhibited delayed insulin responses despite similar plasma glucose levels,^24^ indicating that early β-cell dysfunction is an underlying pathophysiological abnormality of impaired glucose tolerance in this population. Therefore, while it is apparent that South Asians have a higher degree of insulin resistance than do members of other ethnic groups, an early impairment in β-cell function could also be a key pathophysiological mechanism in T2DM development in South Asians.

It has been proposed that early impairment in β-cell function could be the result of intrauterine under nutrition that leads to abnormal pancreatic development.^39^ However, data supporting this hypothesis are inconclusive. A study comparing insulin secretion in 12 insulin-resistant adults who were born with intrauterine growth restriction (IUGR) to 13 controls adults failed to observe any evidence suggesting a defect in insulin secretion in young adults born with IUGR.^40^ Similarly, a study of 8-year-old Indian children noted that lower birth weight was associated with increased insulin resistance but it was not related to reduced β-cell function.^41^ However, a study of middle aged Danes found that participants with low birth weight had elevated concentrations of post-load serum insulin, and after adjustment for insulin sensitivity, also exhibited a reduction in β-cell function.^42^ Therefore, it is possible that intrauterine undernutrition may indeed lead to impairments in pancreatic development, and subsequent β-cell dysfunction, which could be exacerbated by insulin resistance. This could have severe implications for T2DM development in South Asians due to the high prevalence of low birth weight babies born in the region. It is also possible that impaired β-cell function is the result of genetics, epigenetics, or endocrine disruptors, such as pollution. However, the complete mechanistic pathway determining T2DM in this population remains to be elucidated and further studies are warranted.

### Genetics

Genome-wide association (GWA) studies have identified approximately 60 genes associated with T2DM risk.^43^,^44^ However, most of these studies have been conducted in European populations,^43^,^44^ and few studies have attempted to replicate the findings of GWA studies in South Asians.^37^ Two recent replication studies in South Asians determined that common type 2 diabetes variants previously found in European populations, such as PPARG, TCF7L2, FTO, and CDKN2A, are also associated with T2DM in South Asians.^45^,^46^ However, it appears that factors that mediate genetic effects, allele frequencies, or varying polymorphisms may differ between groups for at least some of these genes. The FTO gene, for example, is associated with T2DM in both Europeans and South Asians.^47^–^50^ In Europeans the association is entirely mediated by BMI.^20^ However, in South Asians, the associations among T2DM, FTO, and obesity are inconsistent. While some studies have noted associations between T2DM and FTO that were mediated by obesity,^49^ others have reported associations with FTO variants that were not entirely mediated by BMI, central adiposity, or obesity.^47^,^48^ Furthermore, a recent study from North India reported FTO associations with obesity in South Asians, yet none of the variants tested in this population were associated with T2DM.^51^ The differences in associations among FTO, T2DM, and obesity between South Asians and Europeans suggest ethnic differences in the mechanisms of association between FTO genes and T2DM development. However, there is also considerable heterogeneity as measured by associations among different South Asian populations, which calls for further large scale studies on South Asians of varying cultural, geographic, and economic backgrounds.

Another possibility is that varying genetic polymorphisms could contribute to differences in T2DM risk. Adiponectin is a protein associated with glucose modulation,^52^ and studies have noted lower levels of circulating plasma adiponectin in South Asians, compared with Caucasians.^52^ This difference could be, in part, attributed to polymorphisms in the adiponectin gene, as a study comparing 2,000 normal glucose tolerant patients to 2,000 patients with T2DM reported that a polymorphism of the adiponectin gene is associated with T2DM and obesity in South Asians.^53^ However, this association has yet to be tested in other populations, and thus the functional significance remains unknown.

GWA studies targeting South Asian populations may provide valuable information about novel genetic associations underlying T2DM risk. Recently, a GWA study of T2DM carried out in South Asians found 20 independent single nucleotide polymorphisms associated with T2DM, and common genetic variants were identified at six new loci associated with T2DM in South Asians.^43^ The results of this study indicate that additional genetic associations with T2DM can be made by studying populations of non-European ancestry.

### Age

It is a widely held belief that T2DM onset occurs at a much earlier age in South Asians than in other ethnic populations.^54^ A national survey conducted in India noted that the onset of diabetes occurred before age 50 in 54.1% of cases.^55^ In contrast, in the United States, only 37.6% of cases occurred before age 50.^56^ A longitudinal study of diabetes incidence in Ontario, Canada reported that the median age at diagnosis was lowest among South Asians (49 years), followed by Chinese (55 years), Blacks (57 years), and Whites (58 years).^57^ However, to date there have been no studies directly comparing the age-specific prevalence of T2DM in South Asians living in their home countries to members of other ethnic groups living in the United States or in Europe. There is also a lack of information regarding differences in age-specific prevalence between South Asians living in South Asia and those who have migrated to other countries. Such studies may be able to elucidate whether South Asians are at increased innate susceptibility or whether environmental factors related to migration and nutrition transitions are more at play.

### Anthropometry

While South Asians have lower rates of obesity compared with other ethnic groups, as defined by BMI, they tend to have larger waist measurements and waist to hip ratios, and therefore a greater degree of central obesity. This is seen at any given level of BMI and has been associated with higher plasma insulin levels, increased insulin resistance, and a higher prevalence of T2DM.^58^ A study comparing 12 South Asians and 12 Caucasians matched for age and BMI noted that, compared with Caucasians, South Asians had significantly greater total abdominal and visceral fat.^33^ These results are similar to a previous study comparing South Asian and Caucasian men. Again, despite similar total body fat content, South Asians had greater abdominal adiposity than did Caucasians.^59^ South Asians also exhibit greater abdominal fat as compared to other high-risk ethnic groups. A cross-sectional study examining body size, body composition, and fat distribution among European, Maori, Pacific Island, and South Asian adults reported that South Asians had more total fat, both overall and in the abdominal region, compared with Europeans and Pacific Islanders.^60^

Such differences in body fat composition are seen even in childhood and adolescence. Cross-sectional studies have shown that South Asian children have significantly more body fat than European children, with a greater degree of central fat.^61^ Furthermore, despite having lower birth weight, South Asian babies have more central adiposity than their Caucasian counterparts.^62^,^63^ This predisposition may, in part, be attributed to epigenetic changes in gene expression, resulting from undernutrition related stressors during intrauterine development.^64^ On a global scale, the prevalence of low birth weight is highest in the South Asian region, with India accounting for nearly 40% of global low-birth-weight infants.^65^ Maternal undernutrition during pregnancy is a known cause of low birth weight,^66^ and it is possible that such fetal undernutrition may increase the risk of T2DM development later in life. Prospective studies from India have shown that participants who were underweight as children had a high prevalence of overweight, obesity, and central obesity as young adults.^67^ Additional studies from India have demonstrated that participants with impaired glucose tolerance or T2DM in adulthood had low body mass indices before the age of two, followed by a subsequent early adiposity rebound and then an accelerated increase in BMI through adulthood.^68^ Such studies lend support to the notion that fetal programming *in utero* can promote fat preservation. Furthermore, exposure to excess caloric energy later in life can lead to metabolic imbalances, thus increasing the risk for T2DM in adulthood, as several studies have noted that participants who were small at birth and became obese in childhood have and increased risk of developing insulin resistance.^69^ It is possible that small size at birth can also be predictive of future diabetes development in parents of low-birth-weight babies, as a recent study from Mysore, India noted a linear inverse association between birth weight with both maternal and paternal diabetes development at nine years of follow-up,^70^ thereby indicating possible genetic or epigenetic associations between parental diabetes risk and reduced fetal growth in their offspring.

### Biomarkers

Several biomarkers, such as leptin, adiponectin, C-reactive protein (CRP), and markers of oxidative stress may indicate processes that may influence the increased prevalence of T2DM in South Asians compared to other ethnic groups. Oxidative stress resulting from an increased content of reactive oxygen species (ROS) has been implicated in the parthenogenesis of T2DM development.^71^,^72^ While no studies have been conducted to assess levels of oxidative stress biomarkers in South Asians, it has been shown that oxidative stress has been linked to higher levels of visceral fat, which could place South Asians at an increased risk compared to other ethnic groups.^73^

Adiponectin and leptin are proteins linked with T2DM development. Circulating adiponectin concentrations are decreased in those with T2DM, while leptin levels increase with increasing BMI.^51^,^74^ Associations among adiponectin, leptin and T2DM have been found in South Asians,^51^ and recent studies have demonstrated decreased levels of adiponectin and increased levels of leptin in South Asians compared to Caucasians, despite there being no differences in BMI, waist circumference, or hip circumference.^75^–^77^ Such results highlight the possibility of defects in adipose tissue metabolism in South Asians that extend beyond elevated total abdominal fat.

CRP is a marker of inflammation that is associated with T2DM development in both Western and South Asian populations.^78^,^79^ A study comparing healthy South Asian and European men and women in West London, United Kingdom reported that CRP levels in South Asian women were nearly double that of European women.^78^ A similar study found CRP levels to be 17% higher in healthy South Asian men than in European Caucasians and were associated with greater levels of insulin resistance and central adiposity.^79^ A study from the United States compared CRP levels in 82 South Asian and 55 Caucasian males and reported that on average, Asian Indians had significantly higher plasma CRP levels than did Caucasians.^80^ This difference became even more significant after adjustment for total body fat and waist circumference and suggests an underlying proinflamatory state in South Asians, which could be another important contributing factor toward increased T2DM risk.

## Environmental risk factors for T2DM

### Lifestyle behaviors

In recent decades important demographic and environmental shifts have occurred in South Asia. Life expectancy is increasing while birth rates are declining, resulting in a significant proportion of older individuals.^81^ Additionally, the individual income and per capita expenditure have risen, as has migration from villages to cities.^81^ All of these factors have contributed toward lifestyle changes that now include physical inactivity, a shift away from traditional dietary habits, higher rates of tobacco use, and less sleep.

#### Physical inactivity

It is well established that reductions in physical activity increase the risk of T2DM in all ethnic groups.^82^–^84^ While physical activity levels do not meet recommended guidelines in either Caucasians or South Asians, South Asians appear to be even less physically active than their Caucasian counterparts.^85^,^86^ A systematic review of studies describing levels of physical activity and fitness in UK South Asians identified 12 studies examining physical activity in adults. The differences in physical activity levels in South Asians, compared with general the population, were substantial; South Asian groups reported physical activity levels that were 50–75% lower than those of Europeans.^87^ These results are similar to those from a study analyzing data from the Newcastle Heart Project, indicating that 52% of European men did not meet current guidelines for physical activity, compared with 71% of Asian Indians, 88% of Pakistanis, and 87% of Bangladeshis.^88^

#### Dietary changes

In addition to lower physical activity levels, higher intakes of refined carbohydrates and saturated and *trans* fats have been shown to increase T2DM risk by adversely affecting glucose metabolism and insulin resistance. In contrast, low glycemic index foods and foods high in dietary fiber have been shown to reduce glycemic and insulinemic responses, thereby reducing the risk of T2DM.^89^,^90^ In South India, intake of polished white rice, with a high glycemic index, have been linked to T2DM prevalence after adjusting for potential confounders.^90^ The typical South Asian diet is high in carbohydrates, *trans* fats, and saturated fat.^91^ A comparative study reported higher overall caloric intake, as well as a greater percentage of carbohydrate content in a typical South Asian meal, compared with standard European meals.^92^ Over the past several decade there have been increases in consumption of animal products, sugars, and fats in South Asian diets.^93^ Furthermore, among those who have migrated to Western countries, acculturation has led to a more frequent selection of nontraditional foods.^94^ Specifically, consumption of margarine, juice, chips, colas, alcohol, and fast food has been shown to increase upon migration to Western countries, while consumption of fruits, vegetables, and fiber has been shown to decrease.^95^,^96^ A recent study from Norway reported that after migration, Pakistanis and Sri Lankans increased their consumption of oil, meat, and dairy products, while decreasing their consumption of beans and lentils.^95^ It is also possible that differences in micronutrient intakes could contribute to varying risk in T2DM development. Several micronutrients such as magnesium, calcium, vitamin C, and folate have been thought to play a beneficial role in glucose homeostasis.^97^,^98^ A study from the UK comparing the dietary intakes South Asian, black African-Caribbean, and white European children reported higher total energy intake among South Asian children compared with white Europeans, but lower intake of vitamin C, D, calcium, and iron.^98^ A study assessing dietary intake of Asian Indian adults living in the United States reported insufficient intake of magnesium in males and insufficient intake of calcium in females.^99^ The long-term implications of micronutrient deficiencies in relationship to T2DM in South Asians, compared with other ethnic groups, remains to be elucidated and further studies are required.

#### Tobacco use

Several studies have established the association between cigarette smoking and increased risk for T2DM.^100^–^102^ The association between smokeless tobacco and diabetes risk is less well established.^103^ While tobacco use is a global epidemic, developing countries account for a disproportionate amount of worldwide tobacco consumption.^104^ Recent estimates report that 47% of men over the age of 15 and 14% of women in India are tobacco users,^105^ with a large percentage of users consuming smokeless tobacco products.^106^ For men, the prevalence of tobacco use in India is nearly double that in the United States.^107^ Therefore, comparative studies assessing smoking prevalence and associated T2DM risk across ethnic populations could help determine if excess T2DM risk in South Asians is in part due to greater exposure to tobacco, or if other factors weigh more heavily.

#### Sleep duration

Duration of sleep, both above and below average, has been shown to play a role in T2DM risk. Studies have noted that sleep restriction to only four hours caused decreases in the appetite suppressing hormone leptin and increases in the appetite inducing hormone ghrelin.^108^ In a study of 900 individuals without diabetes followed for up to five years, 146 participants developed incident T2DM. Sleep duration of less than seven hours was a significant predictor of T2DM in Caucasians and Hispanics, even after adjusting for insulin sensitivity and insulin resistance.^109^ In addition to duration, disturbances in sleep have also been shown to be associated with diabetes incidence. A prospective, population based study from Sweden followed nondiabetic participants for approximately 15 years. Study results indicate that difficulties falling asleep or regular use of hypnotics were associated with an increased risk of developing diabetes.^110^ While there have been no studies directly comparing sleep duration or disturbances and diabetes incidence in South Asians and their Western counterparts, a cross-sectional study from India noted a high prevalence of snoring (40%) and daytime sleepiness (59%) in a normal weight urban South Indian population,^111^ both of which showed a significant positive relationship with impaired glucose metabolism. Furthermore, a study examining the associations between sleep apnea and risk factors for metabolic syndrome in North India suggested that obstructive sleep apnea was independently positively associated with fasting insulin levels.^112^ Such associations could be due to neuro–endocrine–metabolic associations related to sleep apnea that also might contribute to the development of T2DM.^113^ Therefore, while it is possible that decreased or increased sleep duration as well as sleep disturbances increase T2DM risk in all ethnic populations, comparative studies are needed in order to determine if sleep duration or disturbance contributes to excess risk for T2DM in South Asians as compared to other ethnic groups.

### Environmental pollutants

Persistent organic pollutants (POPs) encompass a variety of man-made chemicals and are of recent concern with regard to T2DM risk. Several studies in the United States and Europe have noted associations between POP exposure and type 2 diabetes.^114^ A cross-sectional study using U.S. National Health and Examination Survey data reported strong associations between insulin resistance and serum concentrations of POPs.^115^ The findings of this study noted that the association between obesity and diabetes was diminished in people with low POP concentrations. However, the association between obesity and diabetes was strengthened as POP levels in the blood increased. To our knowledge, there have been no studies examining the relationship between POP exposure and diabetes risk in South Asian populations. However, there have been studies noting detectable levels of POP in mussels collected from the coastal waters of India.^116^ High levels of POPs have also been observed in Indian municipal dumping sites.^117^ Given the already increased risk for T2DM in South Asians, it may be useful to examine the association between POP exposure and insulin resistance in cross-sectional and prospective studies conducted on South Asian populations.

## Conclusion

Current evidence suggests that the prevalence of T2DM in South Asians is high and rising both in South Asian countries, as well as in the diaspora. These increases are due, in part, to higher T2DM incidence rates in South Asians compared with Caucasians, which suggests an increased propensity for South Asians to develop the disease. This notion is highlighted by evidence indicating that South Asians (1) are more insulin resistant than Caucasians even at similar levels of BMI and total body fat percent, (2) demonstrate early impairments in β-cell function, (3) exhibit greater tendencies toward visceral fat deposition, even as neonates, and (4) have lower levels of circulating plasma adiponectin and higher levels of plasma leptin.

In addition to possible innate predisposition, South Asians are currently experiencing changes in lifestyle behaviors due to migration or nutritional transitions, resulting in physical inactivity and a shift away from traditional dietary habits to those that include greater overall carbohydrates, saturated, and *trans* fats and lower amounts of dietary fiber. Coupled with an increased propensity for T2DM, the recent shifts in lifestyle behaviors only serve to exacerbate the risk for disease. Future research is needed regarding the etiology and pathophysiology of disease in South Asians, compared with other ethnic groups. Attention should be paid to the complete mechanistic pathway and the relative contributions of both insulin resistance and β-cell function, mechanisms related to early T2DM onset, and variations in genetic polymorphisms and epigenetic processes. Studies focusing on the contributions of tobacco use, sleep duration, and environmental pollutants are also warranted. Furthermore, there is a great need for primary prevention. This need will persist, especially as South Asians continue to become more affluent, have greater access to high-fat foods, adopt more sedentary lifestyles, experience a growing population of aging individuals, and migrate to diaspora countries. Evidence indicates that lifestyle interventions, including increases in physical activity and improvements in dietary quality, are effective at preventing or delaying the development of T2DM in high-risk groups.^118^ While those with polycystic ovarian syndrome, gestational diabetes mellitus, and stress-induced hyperglycemia are all considered at high risk for developing T2DM, evidence suggests that the most cost-effective method for T2DM prevention is to target individuals with prediabetes (fasting glucose between 100 and 125 mg/dL or 2-h post-challenge glucose between 140 and 199 mg/dL).^119^ Results from a population based cohort study indicated that while individuals with prediabetes accounted for 16% of the population, they contribute to over 60% of incident T2DM cases, thereby accounting for a significant proportion of those at high risk.^120^ Two randomized trials in persons with impaired glucose tolerance, The Finish Diabetes Prevention Study and the US Diabetes Prevention Program (DPP), demonstrated that the three year risk of developing T2DM was reduced by 58% in those receiving intensive lifestyle interventions.^121^,^122^ Components in such interventions included lessons on behavior change, physical activity requirement of at least 150 minutes per week, well-balanced diets rich in whole grains, fruit and vegetables, with <30% total fat and no more than 10% saturated fat, and weight loss of at least 5–7%. Randomized controlled trials are now taking place to assess the effectiveness, cost-effectiveness, generalizability, and sustainability of such interventions in South Asians.^123^ If shown to be effective, such preventative efforts need to be directed both at individual and population levels, should be culturally sensitive and aggressive, and should start early in order to reduce the risk of T2DM in this highly susceptible population.
